# Transmutation of MAs and LLFPs with a lead-cooled fast reactor

**DOI:** 10.1038/s41598-023-29002-3

**Published:** 2023-01-30

**Authors:** X. Y. Sun, L. H. Han, X. X. Li, B. L. Hu, W. Luo, L. Liu

**Affiliations:** 1 School of Nuclear Science and Technology, University of South China, Hengyang, 421001 China; 2 School of Mechanical Engineering, University of South China, Hengyang, 421000 China

**Keywords:** Nuclear waste, Nuclear fuel

## Abstract

The management of nuclear wastes has long been a problem that hinders the sustainable and clean utilization of nuclear energy since the advent of nuclear power. These nuclear wastes include minor actinides (MAs: ^237^Np, ^241^Am, ^243^Am, ^244^Cm and ^245^Cm) and long-lived fission products (LLFPs: ^79^Se, ^93^Zr, ^99^Tc, ^107^Pd, ^129^I and ^135^Cs), and yet are hard to be handled. In this work, we propose a scheme that can transmute almost all the MAs and LLFPs with a lead-cooled fast reactor (LFR). In this scheme, the MAs and the LLFPs are loaded to the fuel assembly and the blanket assembly for transmutation, respectively. In order to study the effect of MAs loading on the operation of the core, the neutron flux distribution, spectra, and the *k*_eff_ are further compared with and without MAs loading. Then the LLFPs composition is optimized and the support ratio is obtained to be 1.22 for ^237^Np, 1.63 for ^241^Am, 1.27 for ^243^Am, 1.32 for ^79^Se, 1.53 for ^99^Tc, 1.02 for ^107^Pd, and 1.12 for ^129^I, respectively, indicating that a self-sustained transmutation can be achieved. Accordingly, the transmutation rate of these nuclides was 13.07%/y for ^237^Np, 15.18%/y for ^241^Am, 13.34%/y for ^243^Am, 0.58%/y for ^79^Se, 0.92%/y for ^99^Tc, 1.17%/y for ^107^Pd, 0.56%/y for ^129^I. Our results show that a lead-cooled fast reactor can be potentially used to manage nuclear wastes with high levels of long-lived radioactivity.

## Introduction

Nuclear energy provides almost 10% of electricity production in the world^1^. The use of nuclear energy reduces environmental pollution that can be caused by the use of fossil energy but can lead to another issue, i.e., the management of spent nuclear fuels (SNFs). The latter is becoming a major concern because the minor actinides (MAs: ^237^Np, ^241^Am, ^243^Am, ^244^Cm and ^245^Cm) and the long-lived fission products (LLFPs: ^79^Se, ^93^Zr, ^99^Tc, ^107^Pd, ^129^I and ^135^Cs) will leave behind in the wastes after extraction of U and Pu by PUREX process^2–4^. These LLFPs are considered important in terms of the radiation safety performance of the disposal sites (future exposure dose to the public)^5^.

In addition, MAs are the major toxicity source of the SNFs^6^. It was shown that the potential toxicity can be reduced to 1/100 after 1000 years if effective recovery and transmutation of the MAs can be achieved^7^. Consequently, worldwide researchers are taking action on exploring novel approaches to manage the SNFs.

There have been many studies on the transmutation of MAs and LLFPs in pressurized water reactors^8^, fast reactors^9–13^, accelerator-driven sub-critical systems (ADS)^14,15^ and other systems^16,17^. Particularly, the lead-cooled fast reactor (LFR) produces a fast neutron spectrum which is suitable for transmuting both the LLFPs and MAs. Moreover, LFR has a unique safety advantage over other fast reactors and a lot of attention has been paid to the R&D of LFR^18^.

The feasibility of using reactors or other systems to transmute MAs and LLFPs depends on sufficient neutron excess per fission^19–22^. If an average of approximately 1 wt% MAs is loaded to the LFR core, a transmutation rate of 10% per year or more was foreseen without deterioration of the core characteristics^6^. For LLFPs, an advanced nuclear energy system driven by an intense photoneutron source has been proposed to transmute efficiently the LLFPs assembly composed of ^79^Se, ^93^Zr, ^99^Tc, ^107^Pd, ^129^I, ^135^Cs and ^137^Cs^23^. It is shown that the ^79^Se, ^99^Tc, ^107^Pd, ^129^I and ^137^Cs could be transmuted by more than 30% within 20 years and their effective half-lives can decrease drastically from ~ 10^6^ to less than 10^2^ years.

**Figure 1:**
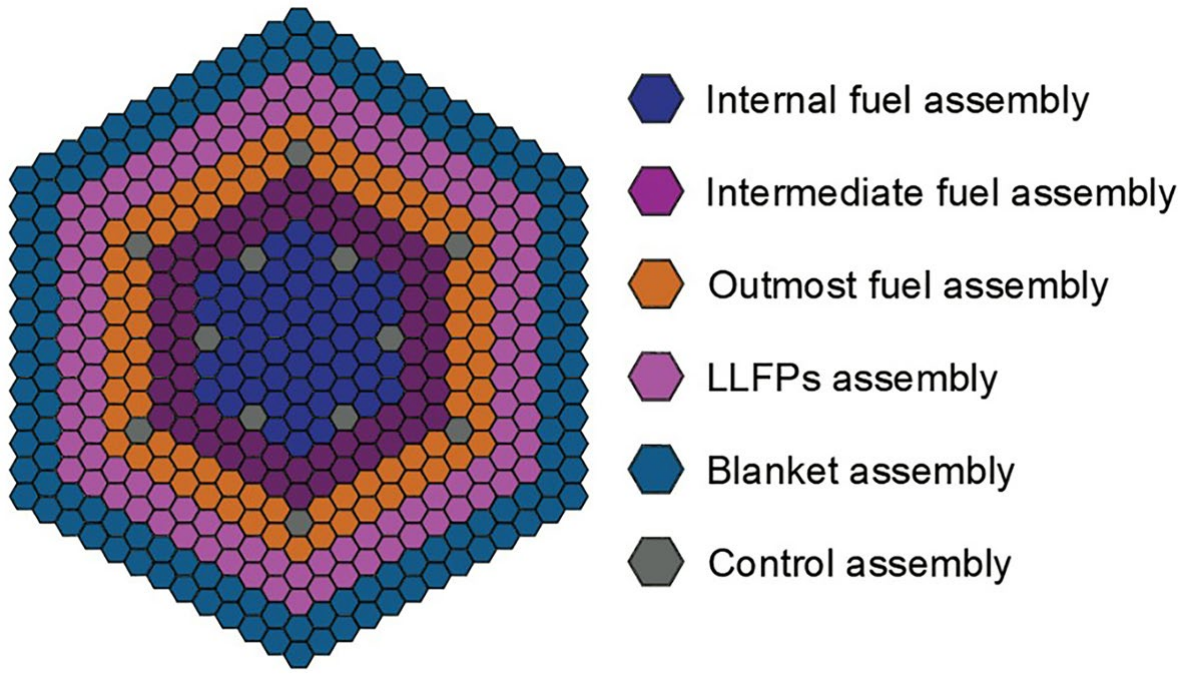
Schematic diagram of the LFR core.

In this study, we propose an LFR core arrangement (see Fig. 1) to transmute simultaneously the MAs and LLFPs. The five MAs nuclides (^237^Np, ^241^Am, ^243^Am, ^244^Cm and ^245^Cm) are loaded in the fuel assembly region with mixed fuel pins, and the six LLFPs nuclides (^79^Se, ^93^Zr, ^99^Tc, ^107^Pd, ^129^I and ^135^Cs) are loaded in the LLFPs assembly region. The loaded mass of MAs and the composition of LLFPs are optimized to balance the LFR characteristics against their support ratio (SR). Then the effect of MAs loaded on the reactor operation is further investigated in the context of *k*_eff_ and neutron flux distribution. The relationship between the transmutation efficiency and the reactor operation time is also discussed. The result shows that the proposed scheme could effectively transmute MAs and LLFPs.

## Methods

### Simulation algorithm

OpenMC code^24^ is an open-source platform for the Monte Carlo simulation of particle transportation, which is spearheaded by the Computational Reactor Physics Group of the Massachusetts Institute of Technology. It uses the continuous-energy cross-section and a constructive solid geometry representation to realize high-fidelity modeling. In our case, we used the ENDF-VIII cross-section library to model the physical processes that occurred in the operation of typical LFRs. The number of total batches is 1200 with each batch taking 10,000 neutron histories. The initial 200 batches were skipped in order to reduce the error caused by source distribution. In the simulations, the neutron energy spectra and the reaction rates are obtained, and the reaction rate has a statistical error of less than 0.05%.

### Core configuration

The layout of the LFR core is shown in Fig. 1. The thermal power of the LFR is designed to be 750 MWt, and the electric power is 300 MWe considering thermal to electric energy conversion of 40%. The LFR core has a height of 1200 mm and a diameter of 3200 mm, which includes 205 Fuel (MAs) assemblies, 114 LLFPs assemblies, 12 control rods, and 138 blanket assemblies. Each fuel assembly consists of 91 pins, which are composed of uranium–plutonium MOX fuel pellets covered by stainless steel cladding. The isotope abundances used for Uranium and Plutonium are listed in Table 1^25^. The LLFPs assembly is comprised of 91 pins, each of which contains one LLFP element; the number of pins containing each LLFP element is balanced to optimize their transmutation. By analogy, each MAs assembly have 91 pins and consisted of MAs pins and fuel pin. The blanket assembly is made of uranium dioxide pellets with a ^235^U enrichment of 0.72%. Note that in the transmutation of LLFPs simulation, we use the “energy-deposition” mode to keep a constant neutron flux over 20 years of operation. The design parameters for the LFR core are detailed in Table 2.

**Table 1:** The isotopic of LFR fuel used in the simulation.

Uranium		Plutonium	
Isotope	Composition (wt%)	Isotope	Composition (wt%)
^234^U	0.004	^238^Pu	2.332
^235^U	0.404	^239^Pu	56.873
^236^U	0.010	^240^Pu	26.997
^238^U	99.583	^241^Pu	6.105
		^242^Pu	7.693

**Table 2:** The main parameters of the LFR used in the simulation.

Main parameters	Value
Thermal power (MWt)	750
Electric power (MWe)	300
Refueling cycle (Day)	600
Core height (mm)	1200
Core diameter (mm)	3200
Number of fuel/MAs assemblies	205
Number of LLFPs assemblies	114
Number of blanket assemblies	120
Number of control rods	12
Fuel assembly at internal area	UO_2_ 84% + PuO_2_ 16%
Fuel assembly at intermediate area	UO_2_ 82% + PuO_2_ 18%
Fuel assembly at outmost area	UO_2_ 78% + PuO_2_ 22%
Number of pins in each assembly	91
Pin diameter (mm)	9.1
Pellet diameter (mm)	10.4

### Selection of LLFPs and Mas

The isotopic composition of LLFPs and MAs used in the simulation is obtained from the depleted fuel of the LFR with a burnup of 30 GWd/t. For the loading MAs, we mainly consider the isotopes ^237^Np, ^241^Am, ^243^Am, ^244^Cm and ^245^Cm. The abundance of loading MAs is listed in Table 3, and all these nuclides are mixed together in a fixed nuclide ratio. Note that ^241^Am and ^243^Am account for more than 85 wt% of the total MAs nuclides, whereas the amount of ^244^Cm and ^245^Cm is only less than 5 wt%. Thus, an efficient transmutation of ^241^Am and ^243^Am is the priority in order to reduce the inventory of MAs. Note that considering that excessive loading of the MAs materials may lead to affect the reactor characteristics^26^, we should optimize the loading amounts of MAs material to enhance the transmutation efficiency while ensuring the smooth operation of core.

**Table 3:** Isotope abundance and half-life of loaded MAs nuclides.

Isotope	Abundance (wt%)	Natural half-life (year)
^237^Np	6.90	2.14 × 10^6^
^241^Am	48.54	4.33 × 10^2^
^243^Am	40.38	7.37 × 10^3^
^244^Cm	4.07	1.76 × 10^1^
^245^Cm	0.10	8.50 × 10^3^

As mentioned above, six radionuclides ^79^Se, ^93^Zr, ^99^Tc, ^107^Pd, ^129^I and ^135^Cs are selected as transmutation candidates. These LLFPs nuclides were mixed with the neutron moderator (70 at% LLFPs + 30 at% YD_2_) and were loaded into the LLFPs assemblies to improve the transmutation performance since the feasibility of this moderator has been proven^9,11,29^. The moderator could soften the neutron at the LLFPs assembly region while having little effect on the neutron energy spectrum of the entire core, as it is loaded in the radial blanket region with a small loading mass. The isotope abundances of these LLFPs nuclides are shown in Table 4, and their chemical forms for assembly loading are explained in detail in our previous work^23^.

**Table 4:** Isotope abundance and half-life of loaded LLFPs nuclides.

Elements	Isotope	Abundance (wt%)	Half-life
Se	^76^Se	0.01	Stable
	^77^Se	1.69	Stable
	^78^Se	4.76	Stable
	^79^Se	10.31	3.25 × 10^5^ a
	^80^Se	22.27	Stable
	^82^Se	60.96	Stable
Zr	^90^Zr	0.22	Stable
	^91^Zr	11.34	Stable
	^92^Zr	15.95	Stable
	^93^Zr	19.79	1.53 × 10^6^ a
	^94^Zr	22.32	Stable
	^95^Zr	3.86	64.032 d
	^96^Zr	26.52	2.0 × 10^19^ a
Tc	^99^Tc	100.00	2.11 × 10^5^ a
Pd	^104^Pd	1.46	Stable
	^105^Pd	39.87	Stable
	^106^Pd	15.31	Stable
	^107^Pd	24.61	6.5 × 10^6^ a
	^108^Pd	18.73	Stable
	^109^Pd	0.02	13.701 h
I	^127^I	23.69	Stable
	^129^I	76.31	1.57 × 10^7^ a
Cs	^133^Cs	31.90	Stable
	^134^Cs	0.66	2.065 a
	^135^Cs	35.80	2.30 × 10^6^ a
	^136^Cs	0.03	13.16 d
	^137^Cs	31.61	30.08 a

### Transmutation rate and support ratio

The transmutation rate (TR) is defined as the ratio of the transmuted amount to the initially loaded one for a specific nuclide in the LFR system, which can be expressed as

1 $$TR=\frac{N\left(0\right)-N(T)}{N(0)T},$$

where *N(0)* and the *T* are the initial atomic number of the nuclide and the irradiation time, respectively. The support ratio (SR) is defined as the ratio of the amount of the transmuted to that of the produced one for a specific nuclide over the same time. Here SR is expressed as

2 $$SR=\frac{N\left(0\right)-N(T)}{YMT},$$

where the *Y* and *M* are the nuclide yield per fission of fuel materials and the total fission rate in the LFR core, respectively. In our study, increasing the mass of initially loaded LLFPs improves SR, and the TR will deteriorate due to neutron self-shielding effects in the loading region. Therefore, comprehensive consideration needs to be taken when evaluating the transmutation efficiency of LLFPs.

## Results and discussions

### Optimization of loaded mass of the Mas

We start with the optimization of the loaded mass of MAs since they could affect the production of delayed neutrons, which play an important role in reactor control. For instance, excessive loading of MAs will deteriorate the LFR operational performance and the neutronic characteristics of the LFR core since the fraction of delayed neutrons produced by MAs nuclides is less than uranium^21,26^. Meanwhile, the LFR core must maintain criticality during the operation, which limits the total loading of MAs nuclides in the core. We select 4 different loading amounts that account for 0.5 wt%, 1.0 wt%, 1.5 wt%, and 2.0 wt% of the total fuel to achieve a high transmutation efficiency. Note that different loading amounts are considered for MAs in our study while their isotopic ratios are fixed (See Table 3).

**Figure 2:**
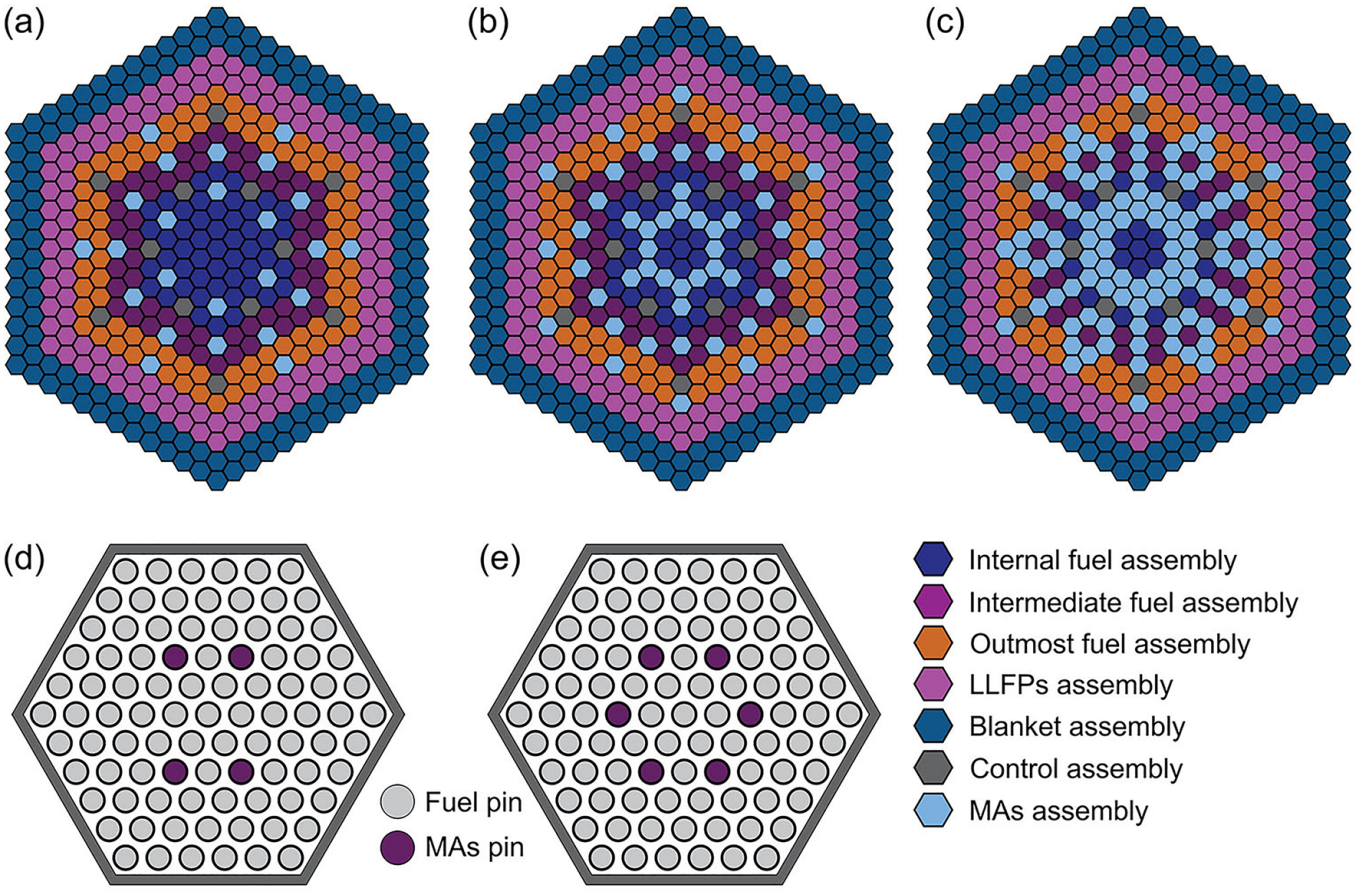
The distribution of MAs assembly in LFR with different loading amounts. In pads (**a**) and (**b**), the loading amounts are 0.5 wt% and 1.0 wt%, respectively and each MAs assembly contains four MAs pins, as shown in pad (**d**). In pads (**b**) and (**c**), the loading amounts are enhanced to 1.5 wt% and 2.0 wt%, respectively, and each MAs assembly contains six MAs pins, as shown in pad (**e**).

The distribution of the MAs pins and assemblies is shown in Fig. 2. The loading mass and the corresponding transmutation for MAs are presented in Table 5. For ^244^Cm and ^245^Cm, their transmutation rate (TR) and SR are negative, which indicates that these MAs cannot be transmuted in this core. However, the impact of ^244^Cm and ^245^Cm on the LFR core is insignificant since their relative fractions in MAs are rather small (< 5.0 wt%). For ^237^Np, ^241^Am, and ^243^Am, these three nuclides are the major transmutation objects since their relative fractions are more than 95 wt%. They can be transmuted by the excess neutron per fission since the TR and SR are positive. In addition, Table 5 indicates that the SR of these nuclides increases with the MAs loaded amount. The SR values are higher than 1.0 when the loaded amount reaches 1.5 wt%. It indicates that a self-sustained transmutation can be achieved, i.e., the transmutation of a long-lived radionuclide exceeds its production during the core operation. The operational performance of the core can be affected by the MAs loading. Therefore, the optimization criterion for the loading of MAs is to achieve self-sustained transmutation of the major MAs with as little loading amount as possible. In the following simulations, the MAs loading amount is set to 1.5 wt%, which can minimize the impact of loading on core performance, as discussed later.

**Table 5:** The transmutation rate and support of MAs considering different loading amounts.

Nuclide	MAs loaded mass (wt%)	Number of MAs pins	Production (g/cycle)	Transmutation (g/cycle)	TR (%/cycle)	SR
^237^Np	0.5	96	1610.88	617.35	12.34	0.38
	1.0	192	1605.22	1320.97	13.19	0.82
	1.5	288	1609.72	1962.09	13.07	1.22
	2.0	384	1615.24	2747.26	13.72	1.71
^241^Am	0.5	96	10,024.54	5175.97	14.41	0.52
	1.0	192	10,030.30	11,002.76	15.32	1.10
	1.5	288	10,035.65	16,360.35	15.18	1.63
	2.0	384	10,045.30	22,804.89	15.88	2.27
^243^Am	0.5	96	9356.28	3786.67	12.67	0.40
	1.0	192	9296.34	8054.14	13.47	0.86
	1.5	288	9229.32	11,969.79	13.34	1.27
	2.0	384	9143.71	16,693.86	13.96	1.78
^244^Cm	0.5	96	1129.10	− 2801.58	− 94.36	− 2.48
	1.0	192	1105.55	− 5937.21	− 99.99	− 5.26
	1.5	288	1083.40	− 8816.30	− 98.98	− 7.81
	2.0	384	1063.38	− 12,253.69	− 103.18	− 10.85
^245^Cm	0.5	96	34.71	− 201.10	− 278.70	− 5.79
	1.0	192	33.33	− 441.62	− 306.02	− 12.72
	1.5	288	32.07	− 648.90	− 299.77	− 18.69
	2.0	384	31.19	− 923.99	− 320.14	− 26.62

### Effects of MAs on the operational performance of LFR

In order to obtain a good transmutation performance, the loaded mass of MAs can be arbitrarily increased. However, the balance between transmutation and operational performance is also required^21^. Generally, the operational performance is sensitive to neutronic characteristics, such as neutron flux and spectral distributions.

**Figure 3:**
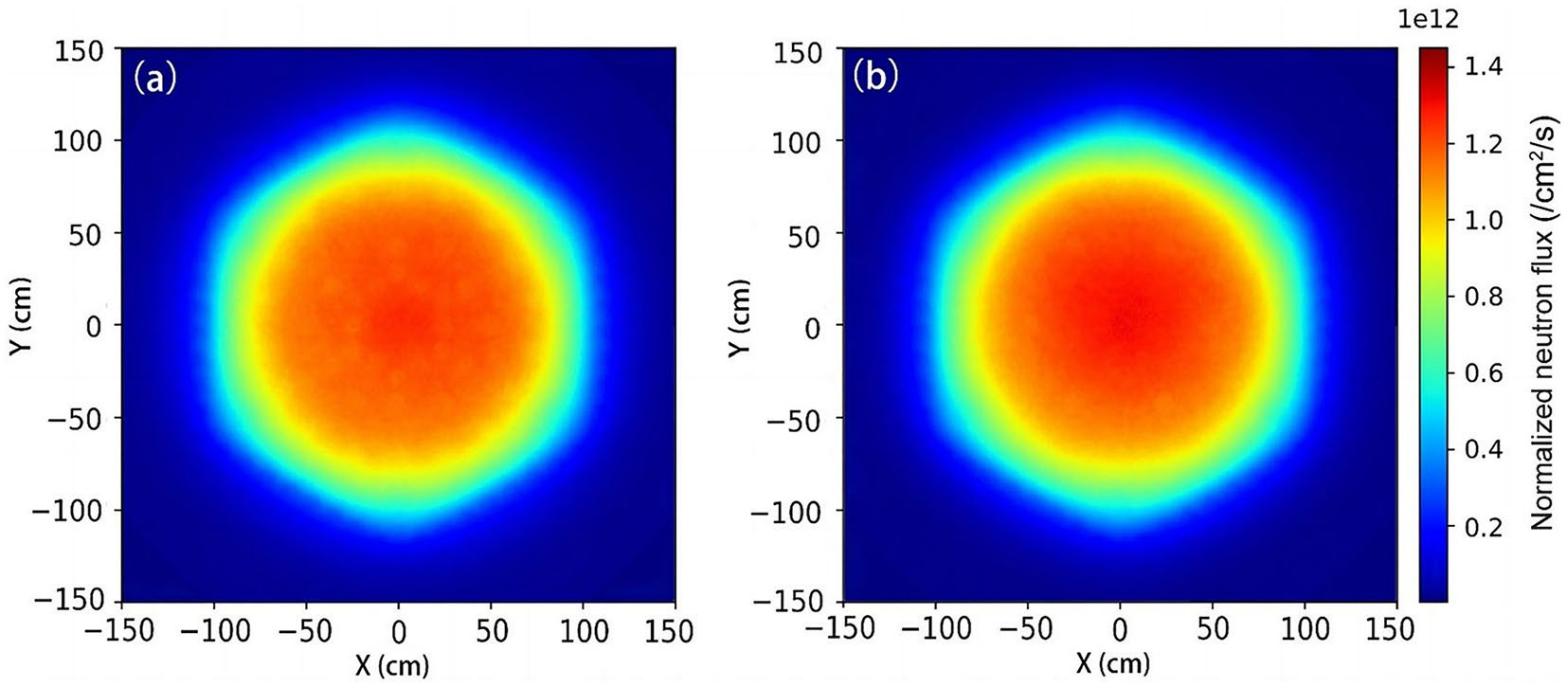
The distribution of neutron flux with MAs (**a**), and without MAs (**b**) at the beginning of the core cycle.

The neutron flux distribution is an important performance characteristic since it could impact lots of many physical parameters including the power peaking factor, which would directly affect the operation of the core. Figure 3 shows the comparison between the neutron flux distributions with and without the 1.5 wt% MAs loaded in the core at the beginning of the fuel cycle. It can be seen that without loading MAs, the neutron flux is peaked at the center of the core and then decreases with the increase of the core radius. With the loading of MAs, the neutron flux also drops with the increase of radius, although it diminishes as seen in the central region compared to the case without loading MAs. We compared the peak fluxes of the two to clarify the differences, and the result shows that the peak flux of the core with 1.5 wt% MAs loaded is 3.98% lower than the case without MAs. Such difference would not significantly deteriorate the physical properties of the core and then affect its operation.

**Figure 4:**
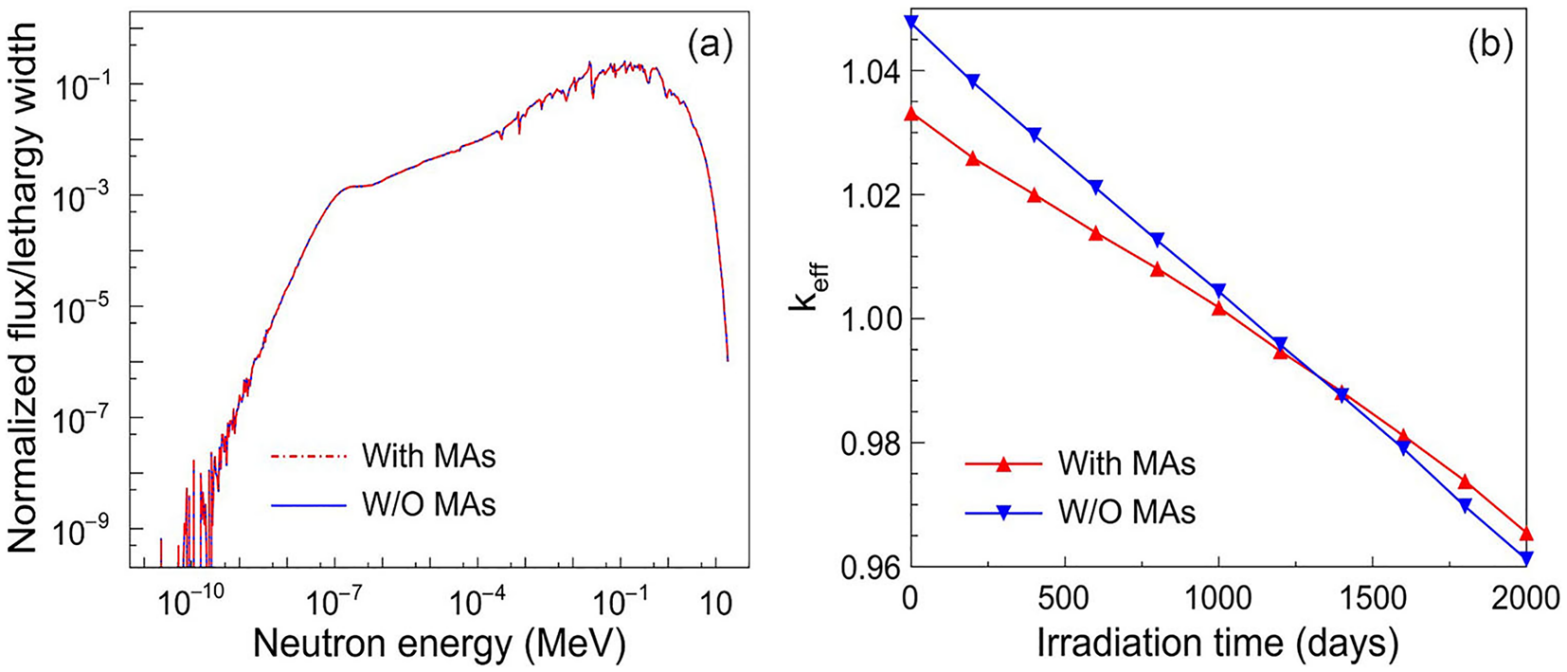
The neutron energy spectrum (**a**) and the evaluation of *k*_eff_ with time (**b**) at the different loading designs (with and without loading MAs).

The neutron spectrum is another important parameter of the LFR, which is related to the Doppler coefficient and power peaking factor^26^. More importantly, the neutron spectrum would significantly affect the fission rate and the transmutation rate. The spectral distributions of neutrons inside the whole core with and without the loaded MAs are shown in Fig. 4a. One can see that the neutron spectrum remains almost unchanged with the 1.5 wt% MAs loaded. It indicates that the loading of MAs would not affect the core operation and nuclear transmutation.

On the other hand, during the operation of LFR, the full power operation time would also influence the transmutation of MAs and LLFPs^6^. Hence, we calculate the time evolution of *k*_eff_ by using the depleted module of OpenMC to investigate the effect of the full power (750 MWth) fuel cycle as adding MAs nuclides to LFR. The corresponding results are shown in Fig. 4b. The *k*_eff_ decreases with operation time no matter whether MAs nuclides are loaded or not. However, the loaded MAs diminish the initial *k*_eff_ and then delays its rate of decline. When irradiation time reaches ~ 1300 days, the *k*_eff_ in the case with MAs loaded is equivalent to that in the case without MAs. As the irradiation time continues to increase, the former one will eventually exceed the latter. This is because the ^237^Np, ^241^Am and ^243^Am will be transmuted to ^238^Pu, ^242^Am and ^244^Am by capturing fast neutrons. These products carrying large fission cross sections could compensate for the reactivity loss^27,28^. The above result shows that the 1.5 wt% MAs loading has an insignificant impact on the operation of the core, and it would not reduce the fuel cycle length.

### Optimization of the LLFPs composition

**Figure 5:**
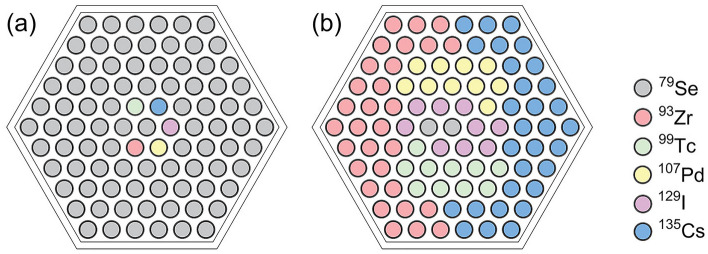
The distribution pattern of pins in LLFPs assembly for the maximum loading of ^79^Se (**a**) and the optimal LLFPs scheme used to balance the transmutation efficiencies of all the LLFPs (**b**).

The transmutation efficiency of LLFPs depends on the neutron capture cross-section and their loading amount. Here we fix the total number of LLFPs pins and adjust the LLFPs composition to investigate the effect of LLFPs loading on the transmutation efficiency. Figure 5a shows an exemplary LLFPs composition in which a maximum loading of ^79^Se is achieved by giving only one pin for each other LLFPs nuclides. Similarly, a maximum loading of ^93^Zr, ^99^Tc, ^107^Pd, ^129^I and ^135^Cs can be realized, respectively. As a result, we obtained six kinds of LLFPs composition schemes. The resulting SR and TR values are shown in Table 6. Here the SR and TR values for the maximum loading are directly obtained, while those for the minimum ones are calculated by averaging the values obtained from the other five LLFPs composition schemes. For ^79^Se and ^93^Zr, the TR values obtained at the minimum loading amount are almost two times smaller than those at the maximum loading amount. However, the opposite results are obtained for the ^99^Tc and ^107^Pd. For ^129^I and ^135^Cs, the TR values obtained at the minimum loading amount are comparable to those at the maximum loading amount. The SR value increases with the loading of all the six LLFPs, which is in agreement with the prediction of Eq. (2). For a minimum loading of ^79^Se, the SR is obtained to be 1.337, indicating that it is easy to achieve self-sustained transmutation. For ^79^Se, ^99^Tc, ^107^Pd, and ^129^I, although their SR values are less than 1.0 in the cases of minimum loading, their SR values are more than 1.0 in the cases of maximum loading. It suggests that the above 4 LLFPs nuclides can realize SR > 1.0 by controlling their loading amount. For ^135^Cs, achieving their self-sustained transmutation is very difficult, because of its neutron capture cross-section.

**Table 6:** The TR and SR values were obtained from different LLFPs composition schemes. The minimum load results are obtained by averaging the TR (or SR) values obtained from the other five LLFPs composition schemes.

Transmutation indicator	Loading amount	^79^Se	^93^Zr	^99^Tc	^107^Pd	^129^I	^135^Cs
TR (%/year)	Minimum	0.62	0.17	1.21	1.52	0.63	0.55
	Maximum	1.22	0.29	0.47	0.57	0.55	0.57
SR	Minimum	0.67	0.01	0.20	0.12	0.14	0.01
	Maximum	122.37	1.03	6.77	3.98	10.90	0.49

In order to achieve the self-sustained transmutation of as many nuclides as possible, a feasible LLFPs scheme is given and is shown in Fig. 5b. In such a scheme, the relative volume ratio of Se, Zr, Tc, Pd, I, and Cs nuclides is set to 2:30:10:10:9:30. Accordingly, the transmutation result is given in Table 7. The SR values of ^79^Se and ^99^Tc exceed 1.0 with a factor of more than 0.3. It indicates that some neutrons might be "wasted" for transmuting only ^79^Se and ^99^Tc, however, they do not act on other nuclides whose SR < 1.0. Note that the transmutation of as many LLFPs nuclides as possible should be achieved in a self-sustained transmutation. Although, both ^93^Zr and ^135^Cs have 30 pins loaded, their SR values are still much less than 1.0. As a result, it is difficult to realize a self-sustained transmutation for these two nuclides by regulating their loaded mass. Other approaches, such as changing neutron moderator and loading method, and employing isotope separation may be useful for enhancing the SR values of the whole system.

**Table 7:** The TR and SR of LLFPs in the LFR (with MAs) using the optimal scheme used to balance the transmutation efficiencies.

Nuclide	^79^Se	^93^Zr	^99^Tc	^107^Pd	^129^I	^n^Cs
TR (%/year)	0.58	0.15	0.92	1.17	0.56	0.49
SR	1.32	0.19	1.53	1.02	1.12	0.15

### Transmutation analysis

**Figure 6:**
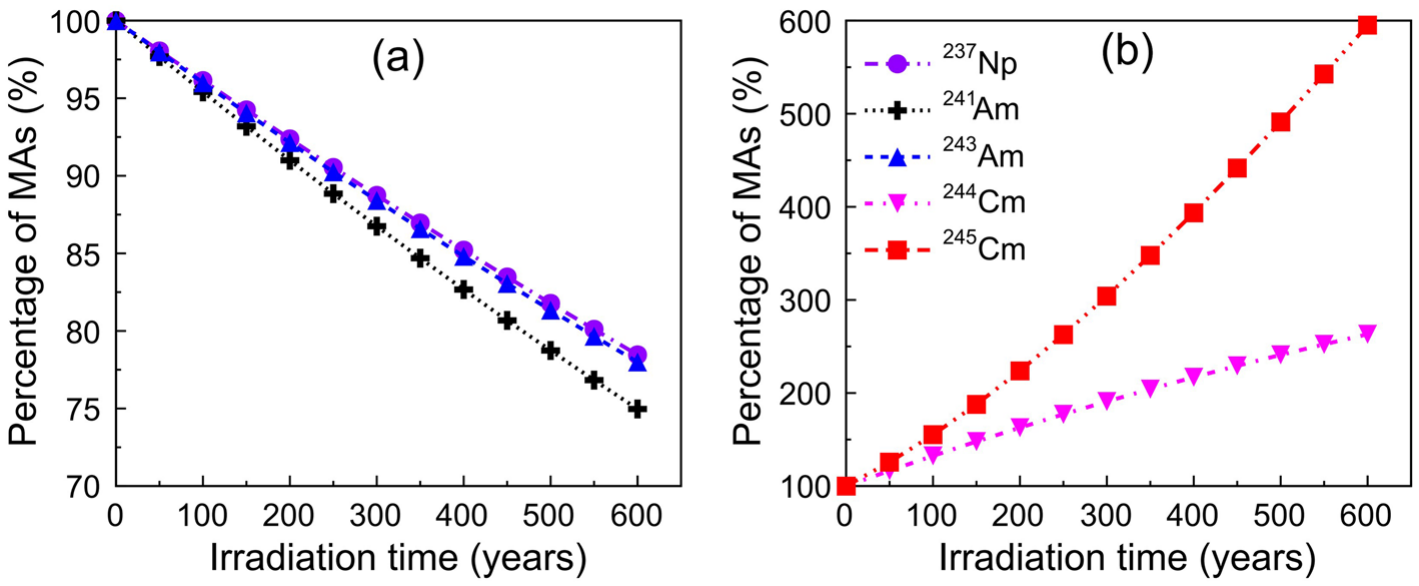
Percentage of MAs remaining in the core over one 600-day cycle. The transmutation percentages for ^237^Np, ^241^Am and ^243^Am are positive (**a**), and the values for ^244^Cm and ^245^Cm are negative (**b**).

This section investigates the effects of transmutation on MA inventories as a function of irradiation time. Figure 6 shows the variation of the transmuted MAs within one cycle. During the 600 days of irradiation, a positive transmutation of ^237^Np, ^241^Am and ^243^Am can be achieved with a transmutation percentage higher than 20%. Among these MAs, ^241^Am has the highest transmutation rate and the transmutation capability of ^237^Np is very close to that of ^243^Am. This is because ^241^Am has the largest fast neutron capture cross-section, whereas those for ^237^Np and ^243^Am are visibly lower. Since the fraction of the above three nuclides accounts for more than 95% of the total MAs nuclides in LFR spent fuel, the present scheme could be utilized to reduce the total inventory of MAs. Different to ^237^Np, ^241^Am and ^243^Am, the mass of ^244^Cm and ^245^Cm increase with time, which means that they cannot be efficiently transmuted by the LFR.

**Figure 7:**
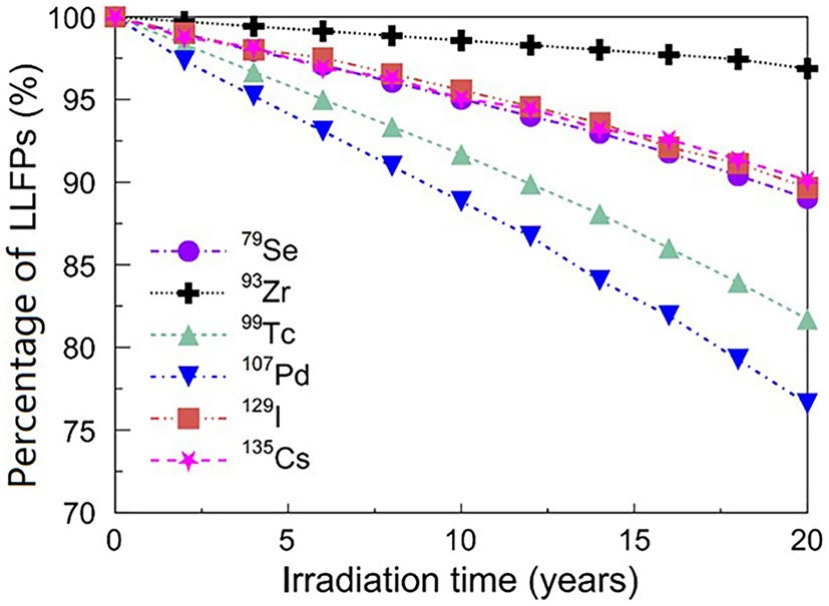
Percentage of LLFPs remaining in the core over a 20-year irradiation time.

The LLFPs transmuted in the blanket regions as a function of irradiation time is shown in Fig. 7. It is found that the transmuted LLFPs increases approximately linearly with the irradiation time. After 20-year irradiation, the percentage of transmuted ^107^Pd and ^99^Tc are higher than 15%, whereas the other nuclides are less than 10%. The transmutation percentage of LLFPs after 20 years are in the order of ^107^Pd > ^99^Tc > ^79^Se ≈ ^129^I ≈ ^137^Cs > ^93^Zr.

## Conclusions

We have presented an LFR-based transmutation of MAs and LLFPs through OpenMC simulations. The loading amounts of MAs are optimized to be 1.5 wt%. The neutron flux distribution and spectrum are chosen as representatives to study the effect of MAs loading on the core operating performance. It indicates that loading an appropriate number of MAs (≤ 1.5 wt%) to the LFR core does not significantly disturb its operation. Moreover, the result of *k*_*eff*_ shows that the refueling cycle is not shortened by the loading of MAs. The transmutation capabilities of MAs and LLFPs are further analyzed by 600 days and 20 years of burnup, receptively. For MAs nuclides, the TR is positive and the SR is more than 1.0 for ^237^Np, ^241^Am, and ^243^Am. Since these MAs account for more than 95 wt% of the total MAs nuclides, the usage of LFR core is expected to reduce the total inventory of MAs. For LLFPs nuclides, The TR is positive for selected six LLFPs nuclides, the SR is more than 1.0 for ^107^Pd, ^79^Se, ^99^Tc and ^129^I. It indicates that all the LLFPs could be transmuted effectively in the blanket assembly, and ^107^Pd, ^79^Se, ^99^Tc and ^129^I could realize self-sustained transmutation. We conclude that the proposed LFR core is helpful to handling long-lived nuclear wastes with high radioactivity. It is worth noting that the loading of MAs has the potential to affect the safety of the core, i. e. the reactivity factor, the value of the control rods, the margin for shutdown, etc., and these works may be the subject of future research.

## Data Availability

The data that support the findings of this study are available from the corresponding author upon reasonable request
